# Bio-inspired 3D printing approach for bonding soft and rigid materials through underextrusion

**DOI:** 10.1038/s41598-024-84525-7

**Published:** 2025-02-05

**Authors:** Arman Goshtasbi, Luca Grignaffini, Ali Sadeghi

**Affiliations:** 1 SDU Soft Robotics, Biorobotics, The Maersk Me-Kinney Moller Institute, University of Southern Denmark (SDU), Odense M, 5230 Denmark; 2 Soft Robotics Laboratory, Department of Biomechanical Engineering, Faculty of Engineering Technology, University of Twente, Enschede, 7522 The Netherlands

**Keywords:** Engineering, Biomedical engineering, Mechanical engineering, Biomimetics

## Abstract

Vertebrate animals benefit from a combination of rigidity for structural support and softness for adaptation. Similarly, integrating rigidity and softness can enhance the versatility of soft robotics. However, the challenges associated with creating durable bonding interfaces between soft and rigid materials have limited the development of hybrid robots. Existing solutions require specialized machinery, such as polyjet 3D printers, which are not commonly available. In response to these challenges, we have developed a 3D printing technique that can be used with almost all commercially available FDM printers. This technique leverages the common issue of underextrusion to create a strong bond between soft and rigid materials. Underextrusion generates a porous structure, similar to fibrous connective tissues, that provides a robust interface with the rigid part through layer fusion, while the porosity enables interlocking with the soft material. Our experiments demonstrated that this method outperforms conventional adhesives commonly used in soft robotics, achieving nearly 200% of the bonding strength in both lap shear and peeling tests. Additionally, we investigated how different porosity levels affect bonding strength. We tested the technique under pressure scenarios critical to soft and hybrid robots and achieved three times more pressure than the current adhesion solution. Finally, we fabricated various hybrid robots using this technique to demonstrate the wide range of capabilities this approach and hybridity can bring to soft robotics.

## Introduction

Animals seamlessly navigate through and interact with their environment thanks to their physical intelligence and adaptive qualities found in natural tissues^1,2^. This inherent capability has created a paradigm shift in robotics design through the emergence of soft robotics, which emulates the characteristics of biological organisms^2,3^. Over the past decade, numerous bio-inspired designs, including the intricacies of an octopus arm^4,5^, the adhesion of a gecko’s leg^6^, the rhythmic motion of an earthworm^7^, and the texture of snakeskin^8^, have emerged, expanding the possibilities for robotics tasks.

Despite the recent emphasis on replicating soft tissues toward developing entirely soft structures^9^, it is crucial to recognize the significant advantages of rigidity in animal biology. For instance, vertebrates’ endoskeletons provide support against gravity and mechanical loads and function as anchor points for soft tissues^10^. Beyond establishing a structural frame, rigidity plays a pivotal role in improving grasping capabilities via claws and nails^11^, providing protective mechanisms, such as the skull and spine safeguarding essential organs and facilitating food grinding through teeth.

Similar to biological structures, integrating rigidity into soft robotic designs can significantly enhance versatility and address the prevalent limitations of these robots^12,13^. For instance, insufficient bending stiffness limits the soft grippers’ tasks involving handling heavy objects and horizontal manipulation- capabilities naturally supported by skeletal structures in animals^14^. Additionally, integrating rigid components can emulate the functional advantages of animal claws and nails, allowing for manipulating both small and large objects^15,16^. Furthermore, the reliance of most soft robots on traditional, delicate electronic components, such as batteries, pumps, and motors, requires protective measures that current soft materials fail to provide^14,17^. Therefore, integrating rigid elements not only compensates for these shortcomings but also significantly broadens the functional scope of soft robotic systems.

While integrating rigid elements in soft robotics design offers a substantial advantage, fabricating such hybrid robots is challenging due to issues in bonding soft and rigid materials^18^. The difference in material properties at the interface can lead to stress concentration and eventual debonding^19–21^. To overcome this, soft roboticists often use adhesives like silicone glue to attach soft and rigid surfaces or connect tubes in pneumatic actuators^22–24^. Although seemingly practical, this bonding method introduces design and repeatability limitations. Furthermore, the reliance on adhesive bonding remains vulnerable, as the adhesive layer often becomes the first point of failure in the robot’s structure.

**Fig. 1:**
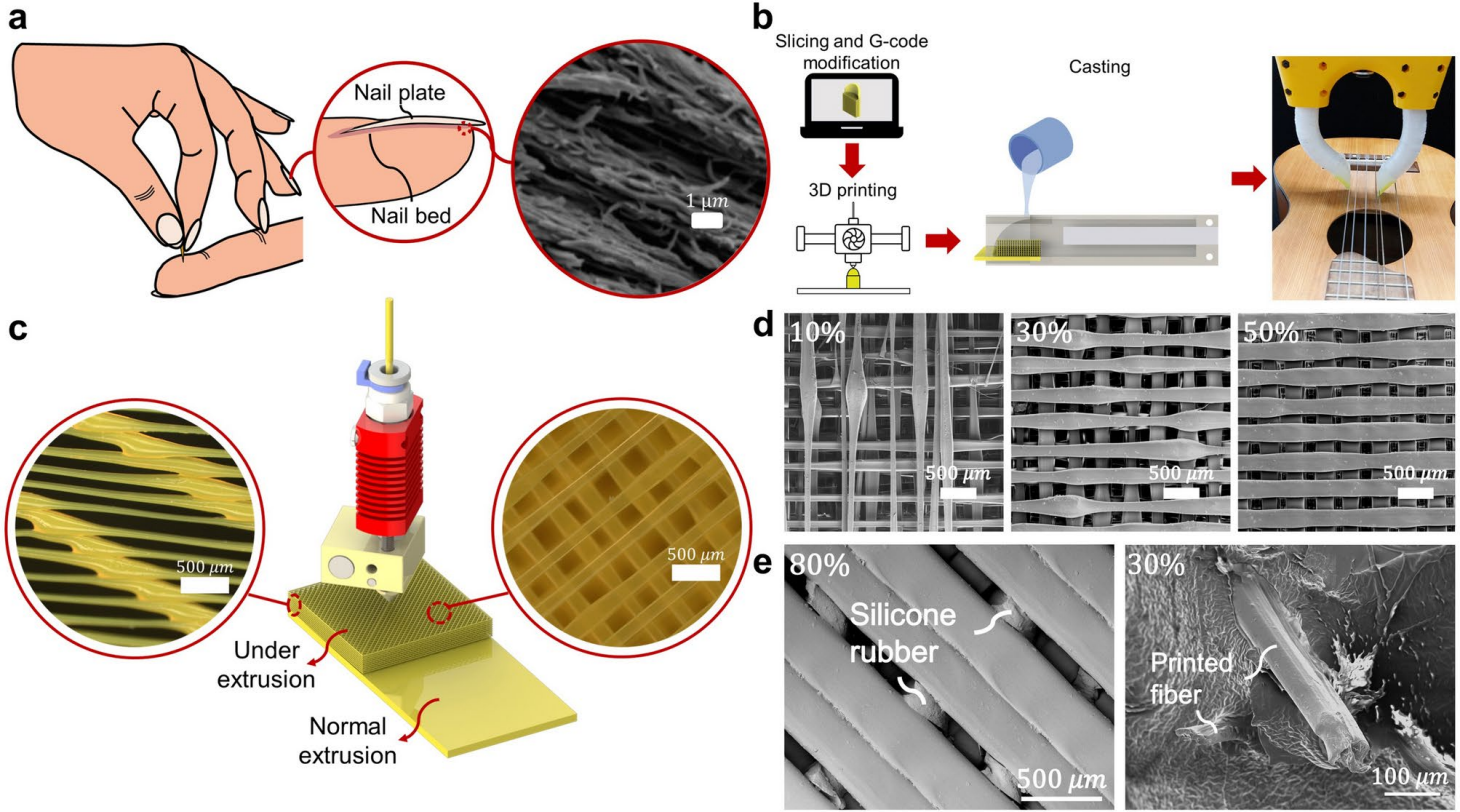
(**a**) In nature, one of the many functions of connective tissue is to firmly bond rigid tissues to softer ones. In the human nail, the nail bed adheres to fibrous mesh keratine filaments at the bottom of the nail plate, shown in (figure reproduced with permission from^34^). (**b**)The procedure followed for the fabrication of a hybrid gripper. (**c**) 3D rendering of a sample used during lap shear bond tests with a zoomed-in microscopic view of the printed fibers. 3D rendering was made with SolidWorks 2021 SP5.1, https://www.solidworks.com/, Dassault Systèmes, France. S/N number 9710-0056-4796-9400. License: University of Twente. (**d**) Scanning Electron Microscopy (SEM) images of samples printed at three different under-extrusion percentages. Conversely, in (**e**), two SEM images of samples printed at different under-extrusion percentages after silicone rubber was cast into the porous segments of the samples. It can be noticed how, at lower flow rates, the soft rubber completely envelops the printed fiber.

In animals’ anatomy, however, rigid and soft tissues are intricately connected through robust yet flexible, fibrous connective tissues^25^. Thanks to their collagen fibers and porous structure, these tissues facilitate a resilient and flexible linkage within the body’s structure. This is achieved through the interweaving of fibers within the tissues^26^ that creates an interlocking mechanism to reduce the stress concentration by creating a stiffness gradient. Similarly, such bonding plays a significant role in human anatomy’s durable connection between soft and rigid structures. For instance, tendons are essential for connecting bones to muscles^27^, Sharpey’s fibers, a matrix of connective tissues, are vital in anchoring the periosteum, the outer layer of bones, to the skin tissues^28^, while the rigid fibrous keratin structure which is the rigid part of nails firmly interwoven with the underlying skin layer, also known as the nail bed, for solid bonding, as shown in Fig. 1a.

So far, increasing the surface of contact has been a technical solution for decreasing the stress concentration using advanced fabricating techniques, mainly additive manufacturing, to achieve more durable bonds between soft and rigid materials^17^. For example, Al-Ketan et al.^29^ and Saldivar et al.^18^ utilized polyjet multi-material additive manufacturing to embed rigidity inside a matrix of soft materials and create a stiffness gradient from soft to rigid for a better interface. Ma et al.^30^ introduced a novel fabrication method combining fused deposition manufacturing (FDM) with material injection. This approach involved 3D printing anchors and hooks directly into a mold, effectively interlocked with the subsequently injected resin, ensuring a secure bond. Additionally, Rossin et al.^31^ suggested another FDM technique to create grids as an interlocking mechanism for soft and rigid materials interface. Another approach uses chemical bonding to bond soft material to rigid material^32,33^.

While these studies present innovative approaches, they have limitations in soft robotics applications. For instance, chemical bonding requires specialized equipment and involves multiple chemical processing steps. Moreover, polyjet multi-material additive manufacturing relies on expensive printers with a limited range of materials. Furthermore, the FDM techniques encounter challenges with the lower density of interlocking points compared to natural fibrous structures, which may affect the durability and effectiveness of the bond in robotics applications.

Therefore, this study introduces an FDM 3D printing technique that utilizes the commonly encountered challenge of oozing to our advantage (Fig. 1). Inspired by^35^, we have developed a method to replicate the complex, fibrous connective tissue structures in nature, creating a more resilient and durable connection between rigid and soft materials. This approach is particularly suited for the commonly used materials in soft robotics. We extensively compare the performance of our technique with traditional adhesive methods, demonstrating superior results in both shear and peeling tests. Additionally, due to the popularity of fluidic actuation and sensing in soft robotic systems, we also examined the airtightness of our bonding method via ballooning test. Our findings indicate that this new method not only overcomes the limitations of silicone glue but also significantly enhances the structural integrity and functionality of bio-inspired robotic systems.

## Results

In the results section, we first examine the microscopy analysis of the underextruded structures. These findings demonstrate how varying levels of underextrusion can effectively control the fiber thickness in the printed material. This insight provides a clear understanding of how precise extrusion settings influence the microstructural characteristics of the fibers. Additionally, we present the results of two mechanical tests, the lap shear test and the peel test, commonly used to evaluate bonding performance. These tests were conducted to investigate the impact of underextrusion on the adhesion between soft and rigid materials. Furthermore, we investigated how well our bonding technique performs in hybrid inflatable structures. This was experimentally proved with a ballooning test. In the case of the hybrid finger demonstrator, we studied the force required to rip off the nail from the soft finger. These results are provided in the Supplementary Material.

The datasets generated and/ or analyzed during the current study are available in the University of Twente, The Soft Robotics Lab, Publication repository, https://tinyurl.com/dk9hxksk.

### Microscope test

The microscopy imaging revealed that the fiber diameter of the porous samples manufactured with the proposed method almost coincided with those predicted by theory Eqs. (1–5). As presented in Table 1, the mean absolute error before predicted and experimental values of the diameter of the fibers is approximately 7 $\upmu$m, with the sample printed at 100% showing the most significant deviation of 12 $\upmu$m.

**Table 1:** Results of the microscopy measurements for samples printed at different flow rate percentages. The measured diameter of the PLA fibers is compared with the value predicted in Eq. (5) in the methods section. The last row is the percentage of the porosity level measured by weight.

Flow rate percentage (%)	Predicted diameter ($\upmu$m)	Measured diameter ($\upmu$m)	Absolute error ($\upmu$m)	Porosity (%)
10	82	73	9	89.22
20	122	123	1	79.51
30	162	164	2	69.40
40	202	193	9	60.24
50	242	252	10	49.14
60	282	277	5	39.87
80	362	366	4	20.26
100	442	430	12	0

### Bonding tests

**Fig. 2:**
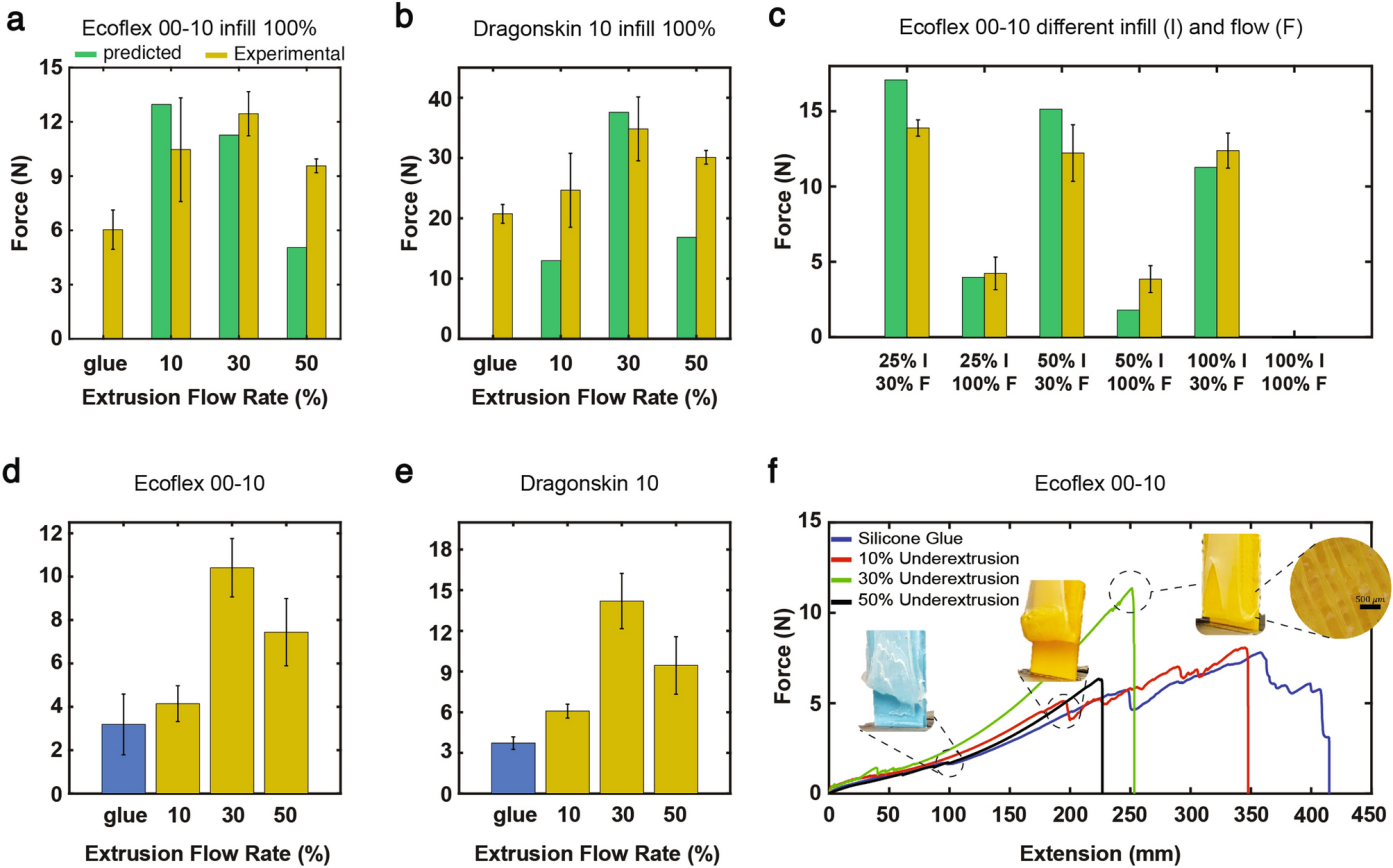
Lap shear and peel-off test results. (**a**, **b**) Theoretical model predictions and experimental debonding forces for Ecoflex 00-10 and Dragonskin 10, respectively, with varying extrusion percentages and Sil-Poxy bonding. (**c**) Effect of infill density on the debonding forces for samples printed with underextrusion (i.e., 30% flow) and with 100% flow showing alignment between theoretical and experimental values. (**d**, **e**) Peel-off test results for PLA-Ecoflex 00-10 and Dragonskin 10, comparing Sil-Poxy (blue) with 10%, 30%, and 50% underextrusion (yellow). The values reported in the peel-off test bar plots referred to the initiation of breakage of the hybrid samples at the interface section of the free silicone and the bonding junction. (**f**) For Sil-Poxy and 10% underextrusion, the Ecoflex 00-10 strip detached completely, requiring greater extension and force before rupture. (The raw data can be found in data availability).

All the experimental results of the lap shear tests conducted in this study are compared to the results offered by the theoretical model proposed in the “Methods” section. The lap shear test experimentally proved that our proposed technique offers a better bonding solution when manufacturing hybrid structures compared to commercially available adhesives such as silicone glue. As illustrated in Fig. 2a,b,d,e, all the different specimens made with our under-extrusion technique recorded higher values of the debonding force than the used silicone adhesive in both lap shear and 180 peeling test for both Ecoflex 00-10 and DragonSkin 10. For Ecoflex 00-10, the model predicted the maximum debonding forces for the samples printed at 10% (i.e., 21.10 N). However, they recorded 10.46 ± 2.87 N at break. it was shown experimentally that the samples made at 30% showed maximum values of 12.45 ± 1.22 N, compared to a predicted value of 11.26 N. The samples made at 50% respectively recorded 9.57 ± 0.38 N in the lap shear test, against a predicted value of 5.09 N. The samples attached with the glue all showed an early onset of debonding, as they recorded much lower values of debonding forces at 6.03 ± 1.08 N. When using DragonSkin 10, the model predicted the maximum debonding forces for the samples printed at 30% (i.e., 37.57 N). This is in accordance with the experimental results, as the 30% samples offered the best bonding solution with recorded values of 34.82 ± 5.29 N. The 10% samples recorded debonding forces of 24.64 ± 6.13 N (compared to a predicted value of 23.76 N). In comparison, the 50% samples registered 30.11 ± 1.12 N (compared to the 16.83 N anticipated by the theoretical model). For the infill percentage test, the samples printed with the proposed technique showed higher debonding strength for all three infill percentages, both theoretically and experimentally, when compared to the samples printed at an extrusion rate of 100%, as demonstrated in Fig. 2c. The 25% infill with 30% flow recorded the maximum average forces (i.e., 13.88 ± 0.54 N) in accordance with maximal values of the predicted force (i.e., 17.07 N). On the other hand, the 50% infill with 30% flow samples recorded debonding forces of 12.21 ±1.87 N (compared to a predicted value of 15.13 N), while the results of the 100% infill at 30% flow match with the ones presented for the other lap shear test. When the PLA samples were printed at 100% infill and with the recommended flow of 100%, the silicone rubber was not able to penetrate into the porosity, and the test samples broke while being detached from the mold. Because of this, we assume a debonding force of 0 N by the predicted value of 0 N. The 50% infill with 100% flow samples recorded debonding forces of 3.84 ± 0.89 N (compared to a predicted value of 1.79 N), while at 25% infill and 100% flow, the samples debonded at 4.23 ± 1.08 N (compared to a theoretical force of 3.97 N). The peel-off test proved further that the proposed manufacturing solution offers a better bonding solution than the primer. When bonded to Ecoflex 00-10, the samples made at 30% showed maximum values of 10.41 ± 1.35 N. The samples made at 10% and 50% respectively recorded 4.14 ± 0.82 N and 7.44 ± 1.55 N, respectively. The samples attached with Sil-Poxy recorded much lower values of debonding forces at 3.18 ± 1.39 N in the peel-off test. When using DragonSkin 10, the 30% samples still offered the best bonding solution, as they recorded values of 14.20 ± 2.03 N. The 10% samples recorded debonding forces of 6.09 ± 0.51 N, while the 50% samples registered 9.45 ± 2.12 N. On the other hand, the samples bonded with Sil-Poxy silicone required much lower forces to detach from the PLA substrate (i.e., 3.72 ± 0.46 N).

**Fig. 3:**
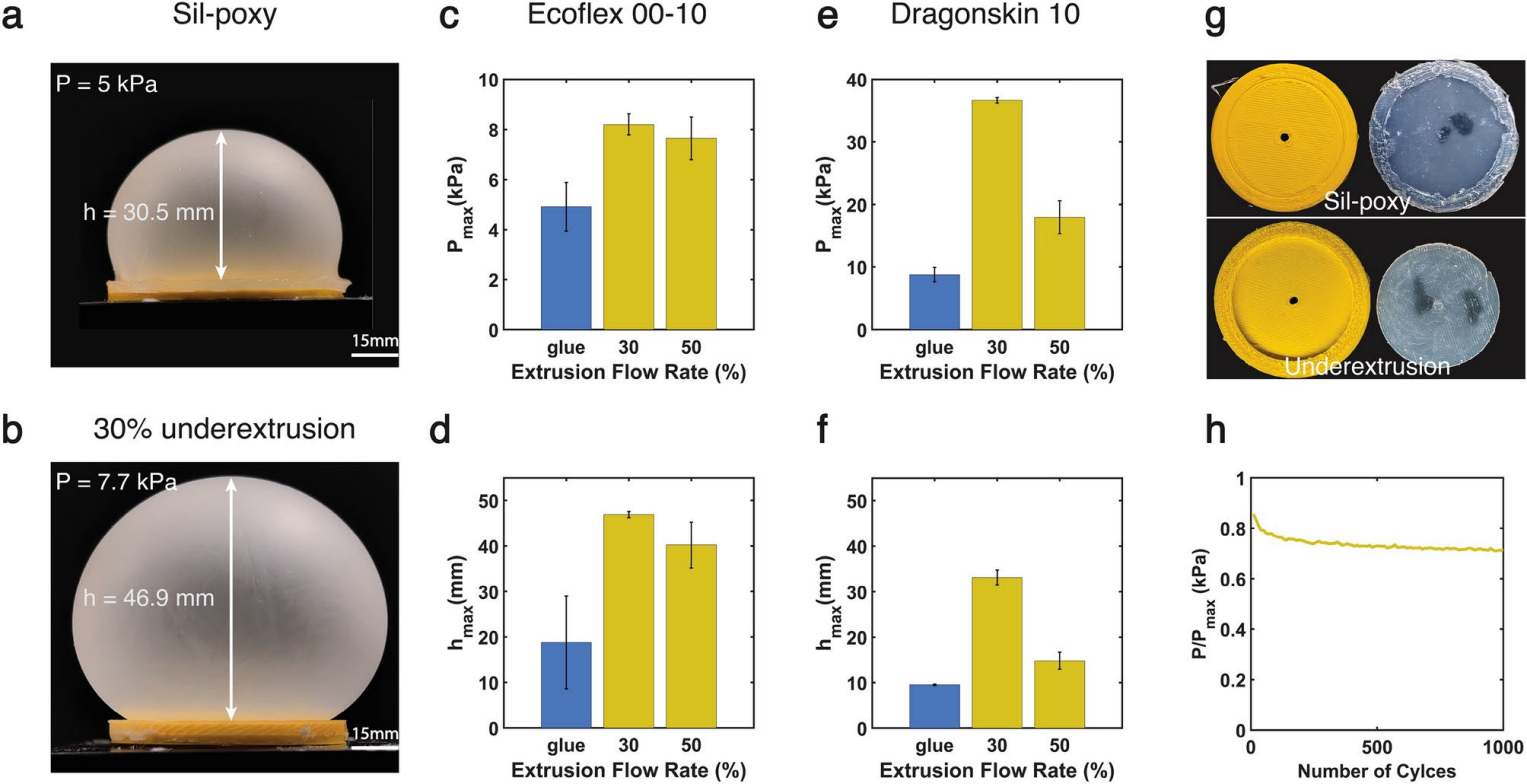
Ballooning pressure test results for hybrid inflatable samples bonded with glue and underextrusion. (**a**) Ecoflex 00-10 with Sil-poxy before failure and leakage. (**b**) Ecoflex bonded with underextrusion, showing plastic deformation without rupture while withstanding higher pressure (7.7 kPa) than glue. (**c**, **d**) Maximum pressure and deflection of Ecoflex 00-10 for three bonding methods. (**e**, **f**) Maximum pressure and deflection of Dragonskin10 for two underextrusion rates and Sil-Poxy. (**g**) Ruptured Sil-Poxy and underextrusion samples showed failure due to adhesive bond detachment (glue) and stress concentrations at the interface (underextrusion). (**h**) The Ecoflex 00-10 cyclic test bonded to PLA with 30% underextrusion for 1000 cycles and 80% of the maximum value of highest pressure from (**c**) (The raw data can be found in data availability).

### Pressure test

As detailed in the Experimental Setup and Materials section, given the prominence of pneumatics in soft robotics, we focused on the effect of underextrusion on the bonding strength between PLA and a layer of silicone rubber during pressure-induced expansion. As presented in Fig. 3, the findings indicate a significant increase in the pressure threshold and expansion peak for samples subjected to underextrusion compared to samples joined by adhesive for both Ecoflex 00-10 and Dragonskin 10.

During the Ecoflex 00-10 experiments, the pressure and expansion values of the underextruded samples (both 30% and 50%) exceeded those of the Sil-poxy bonded samples. As shown in Fig. 3c, pressures of 8.2 ± 0.4 kPa and 7.6 ± 0.8 kPa were achieved for 30% and 50% underextrusion, respectively, compared to 5 kPa for Sil-poxy. Additionally, we observed peak expansion points of 46.90 ± 0.66mm and 40.22 ± 6.84mm, respectively, leading to plastic deformation of the elastomer while maintaining bond integrity.

In the bonding test with Dragonskin 10, the 50% underextrusion sample reached a pressure value of 18.0 ± 2.6 kPa, while the 30% underextrusion sample reached 36.6 ± 0.4 kPa. The adhesive-bonded sample, however, withstood pressures below 8 kPa, indicating significantly lower pressure tolerance. Moreover, the peak expansion observed for the 30% and 50% underextruded samples were 33.14 ± 1.63 mm and 14.82 ± 1.90mm, respectively, surpassing the adhesive sample, which was limited to 9.54 ± 1.56mm (Fig. 3e,f).

Finally, as shown in Fig. 3h, the Ecoflex 00-10 samples bonded via 30% underextrusion tolerated 80% of the maximum pressure for 1000 cycles, with a slight pressure drop from 6.7 to 5.7 kPa due to the plastic deformation of the silicone membrane.

## Discussion

### Validation of the technique

#### Microscopy

Microscopic imaging showed that the widths of the extruded filaments made with the proposed technique follow the ones predicted by Eq. (5), as the computed average error is approximately 8.8 $\upmu$m. The deviation observed in the measured fiber widths compared to theoretical values may stem from various factors associated with the 3D printer itself. A dirty or partially clogged nozzle, along with an incorrect alignment of the printer’s z-axis or of the printing bed, are all factors that can influence this result and, therefore, affect the repeatability of the proposed technique. The collagen fibers that comprise the connective tissue of animals in nature are characterized by an average diameter ranging between 1 and 10 $\upmu$m^36^. Using our method, we obtained fibers with a minimum average diameter of 73 $\upmu$m by deliberately underextruding at a 10% flow rate. Although reproducing the fibers of connective tissue found in nature was not our research’s main goal, we believe this could be possible with our technique by further tuning the printing parameters. Additionally, we can see that by reducing the flow rate percentage percentage at constant printing speed (i.e., 80 mm/s), there is an increasing number of irregularities in different sections of the extruded filament, which is characterized by the alternation of thin sections with more pronounced bulges, as shown in the SEM images in Fig. 1d, and in the optical microscopy images shown in Fig. S1 of the Supplementary Material. This phenomenon could be caused by a physical phenomenon typically occurring in viscoelastic fluids, known as the Plateau-Rayleigh instability^37^. By reducing the flow rate percentage, the surface tension forces prevail over the viscous forces, forming more significant instabilities in the extruded fibers. Another cause for the generation of these instabilities is strictly machine-related. Decreasing the flow rate reduced the amount of material by a factor $\gamma$, as shown in Eq. (4). However, decreasing the flow rate does not affect the print speed, also known as the feed rate, which was kept at 80 mm/s for printing all the microscopy samples. The relatively high feed rate compared to the low flow rates causes stretching and thinning of the print filament, resulting in more extensive and more pronounced instabilities, and it is defined as “glob-stretch printing” in^35^. Therefore, the feed rate directly affects the quality and the homogeneity of printed fibers, as samples printed at lower speeds present more consistent and homogeneous sections along the printing direction. This was confirmed through optical microscope analysis of fibers from samples printed at a constant extrusion rate (i.e., 30%) and at three different speeds (i.e., 20, 40, and 80 mm/s). However, this does not seem to affect the overall fiber diameter, as similar values were measured in all the samples with minimal variation. Despite this result, we believe this trade-off between fiber quality and print speed should require further investigation..

All the test samples showed proper penetration of silicone rubber inside of the porosity, as proven by the illustrated in the SEM images shown in Fig. 1e,f (Supplementary Fig S2), and in the optical microscopy snapshots in Fig. S1 in the supplementary material. By decreasing the printing flow rate, which translates into a higher grade of porosity, a more significant amount of rubber can penetrate the porous segment. For instance, the silicone can completely envelop and surround the fibers printed at 30%, as shown in Fig. 1f. In contrast, the porous sample printed at 80% flow rate shows visible gaps between the portions of the silicone rubber, as shown in Fig. 1e.

#### Bond tests

After performing the lap shear and peel-off tests, we demonstrated that our proposed method offers an alternative bonding solution between rigid materials and silicone rubber to already existing techniques^31^. We experimentally proved that our technique significantly outperforms commercially available alternatives such as silicone rubber glue. Overall, the use of under-extrusion as a bonding technique shows to prevail over the silicone adhesive in both tests, with the 30% flow rate showing the most considerable improvement (i.e., 106.2% and 226.5% in the lap shear and peeling tests for Ecoflex 00-10, and 68% and 281% for Dragon Skin 10, respectively) from the latter. In the case of the lap shear test, which is one of the most popular tests to study the bonding between different materials, we present a theoretical model that allows the estimation of the debonding forces for our systems under different 3D printing conditions. The model can be found in more detail in the methods section. Despite the model providing a very simplified situation of the actual test conditions, where all the samples are made with no manufacturing flaws and imperfections, and the hyperelastic behavior of the rubber is not taken into account, it was able to accurately predict the modality of failure of all the samples that occurred experimentally for both Ecoflex 00-10 and DragonSkin 10. In the samples that were printed at flow rates higher than 10%, failure occurred due to the rupture of the silicone itself in correspondence with the materials section of the interface. As previously mentioned, increasing the extrusion rate results in printed fibers of larger dimensions, reducing the rubber occupied by each section of the porous segment. This ultimately reduces the value of the maximum force introduced in Eq. (6), thus leading to an early onset of failure. On the other hand, the entire silicone rubber strip that was still containing the porous PLA was completely ripped off in the samples printed at 10%. In these cases, where the printed fibers are very thin (i.e., layer width < 100 $\upmu$m), failure occurs theoretically due to the rupture under shear of the vertical beams that connect the infill lines with the external wall, as the force that is needed to break these beams (see Eq. 8) is lower than the one required to break the silicone rubber under pure tension. The exact failure mechanisms were experimentally observed in the peeling tests for both Ecoflex 00-10 and DragonSkin 10. The proposed theoretical model predicted the highest debonding force for the samples at a 10% extrusion rate. However, the average debonding forces recorded during the experiments were approximately half the predicted value. As previously mentioned, the model was based on ideal samples without any inconsistencies in the porous segments. Nevertheless, when printing at lower extrusion rates, such as 10%, there is a higher probability of having fiber irregularities, thus affecting the repeatability of the print. Not only could this lead to beams of different diameters across the sample, thus further reducing the debonding force, but it also explains the higher standard deviation encountered during the experiments. The predicted values of the debonding forces for the samples printed at 30% flow rate were close to experimental evidence across all tests for both silicone rubbers, while at 50%, the experimental values almost doubled the predicted forces. This can be explained by an inaccurate estimation of the layer height shrinking coefficient presented in Eq. (8) of the supplementary material, which was done based on experimental evidence of samples that were printed at a 30% flow rate. Such function should require thorough investigation, as more experimental proof at different flow rates should be collected for data fitting and optimization of the proposed model. We believe that an advantage of the proposed underextrusion technique compared to other bonding methods (e.g., the method proposed in^18^) is that it can be used at any infill percentage, while samples printed at 100% flow require lower infill density to achieve interlocking between the two materials. Conversely, to the samples printed with our method, the specimens at 25% and 50% infill densities and 100% extrusion rate were printed without any walls in order to achieve proper bonding between the two materials. This design variation slightly changes the theoretical model, as explained in the methods section. Despite this change, the samples bonded through underextrusion with the external wall still achieved larger predicted and experimental forces than the samples at 100% flow at any infill percentage. The predictability of the debonding forces, even after these modifications, proves that the model could possibly be adapted to different geometries and infill patterns, which will be investigated in future work. Furthermore, the addition of underextrusion to high infill percentages leads to a larger number of printed fibers and unit cells inside of the porous segment, which leads to a reduction of the volume of silicone rubber penetrating the sample. Although this reduces both theoretically and experimentally the overall debonding forces, as proven by the lap-shear infill test results shown in Fig. 2 c, this allows the achievement of smaller bonding interfaces in small-scale hybrid robotic applications. Conversely, hybrid parts that require wider bonding areas of interface could benefit more from lower infill percentages. Furthermore, it is essential to clarify that the values of the reported forces in the peeling test (see Fig. 2) d and e relate to the instances where the samples that were printed with an underextrusion percentage larger than 10% broke, as it equaled the onset of failure of all the samples. This choice was made as in most soft robotics applications (fluidic based), the first failures of the system occur with the onset leakage points, which may cause the robotic system as a whole to fail its purpose. We believe that the initial failures of the peeling tests in the proximity of the bonding interface section between the two materials can be classified as a potentially detrimental leaking point. In reality, the glued and 10% samples recorded higher forces at their complete rupture, as shown in Fig. 2f, which is logical considering the higher extension required to pull the entire silicone band. We believe that this method not only offers an easily reproducible bonding technique on its own but that it could also be integrated as part of other existing methods such as the one presented in^18,31^, in order to further increase their performance.

#### Pressure test

The Underextrusion 30% bonding outperformed the Sil-poxy bonding for both Ecoflex 00-10 and Dragonskin 10, showing similar results to previous bonding tests. As presented in Fig. 3, for Ecoflex, we achieved 164% more pressure and 249% more maximum deformation. Moreover, we observed that both Underextrusion 30% and Underextrusion 50% reached plastic deformation. Underextrusion 30% withstood the maximum possible air volume of the syringe system without any rupture, while Sil-poxy failed before reaching the plastic point (Video S3 in the Supplementary). The cyclic results in Fig. 3h indicate that despite the transient phase of the recorded pressure, the Underextrusion 30% bonding is durable and resilient under cyclic loads. The pressure drop in the test is due to slight plastic deformation of the Ecoflex membrane.

For the Dragonskin membrane, we achieved a more significant pressure difference (4.5 times) and maximum deformation (3.5 times) between Underextrusion 30% and Sil-poxy bonding. One main reason for this considerable pressure difference is the limited pressure tolerance of Sil-poxy in both Ecoflex and Dragonskin. In contrast, Underextrusion bonding allows Ecoflex to reach plastic deformation at pressures close to Sil-poxy’s failure point, thus minimizing the pressure difference between the samples.

Finally, the reason that 30% outperforms 50% is similar to the bonding tests: the high density of fibers in the 50% porosity prevents the silicone from penetrating properly inside the structure. This result is more significant when comparing the Ecoflex 00-10 and Dragonskin 10 samples, where the differences between 30 and 50% are smaller for Ecoflex 00-10 due to its lower viscosity, allowing easier penetration. Furthermore, in underextrusion cases, ballooning failures occur at the connection points between the non-bonded silicone section and the 3D-printed part, similar to the bonding tests. This failure is due to stress concentration from the rigid underextrusion to the soft silicone rubber. This is another reason why 30% underextrusion performs better than 50%, as the transition is smoother due to the lower stiffness of 30% underextrusion.

### Hybrid grippers

**Fig. 4:**
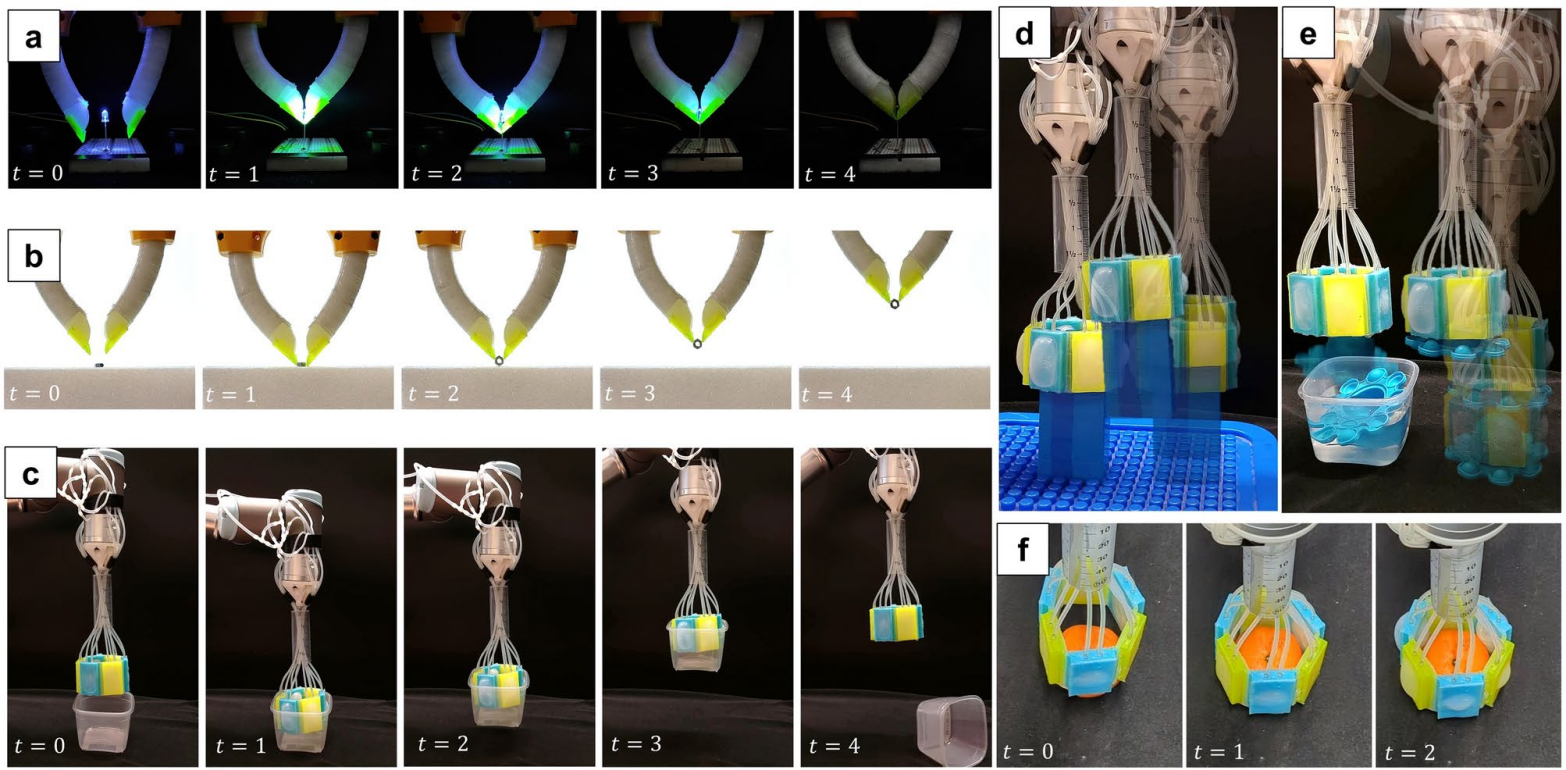
Hybrid grippers manufactured using the proposed technique. A bioinspired hybrid gripper mimicking a human fingernail features a rigid nail plate bonded to a porous mesh, replicating the natural bond between a nail and nail bed. This design enables the manipulation of small objects like an LED (**a**) or an M2 nut (**b**). A hybrid (soft and rigid) hexagonal inflatable gripper is demonstrated. It can manipulate objects using its outer surface (**c**) and inner sides (**d**, **e**). Both inflatable membranes and soft hinges, bonded to rigid segments through underextrusion bonding, enhance adaptability for gripping objects of varying dimensions. (**f**) The technical drawings for these grippers are available in Supplementary Material Figs. S9 and S10.

Two hybrid grippers were designed and made for this project using our proposed technique to secure bonding between rigid and soft materials. Inspired by the dense connective tissues found in vertebrates, we reproduced its interwoven fibrous structure using the proposed technique to connect a soft bending actuator to a rigid nail, thus recreating the bonding between the human nail and its underlying soft skin layer. One of the key limitations of conventional soft grippers, such as fiber-reinforced bending actuators, is their difficulty in picking up small objects. In a manner similar to how humans use their nails to grasp tiny items, incorporating a “nail” element into the design of a hybrid gripper enables it to handle a broader range of small objects, such as LEDs and nuts, as demonstrated in Fig. 4a,b. Furthermore, since the nail is attached to the outer layer of the actuator, it does not affect the overall performance of the actuator. As proven by the ballooning pressure tests, our method provides a bonding solution when fabricating inflatable structures, as it is able to withstand higher pressures when compared to other alternatives, such as silicone glue. Therefore, this can be exploited, for instance, when designing hybrid inflatable systems such as the gripper shown in Fig. 4c–f. The gripper is characterized by two different inflatable surfaces, which can be actuated simultaneously or separately, thus allowing to pick objects from both sides, as shown in Fig. 4c–f. The gripper is not only able to pick up objects of different shapes and sizes thanks to the softness of its inflatable paddings, but its rigid body provides a structural integrity that allows it to lift heavier objects. Furthermore, thanks to its excellent bonding properties under tensile loads, the proposed technique was also utilized in designing the soft joints that connect the different taxels of the gripper together. The use of soft joints confers great adaptability to the hybrid gripper, thus allowing it to adjust to objects of different shapes without rupture, as shown in Fig. 4d. (Supplementary video S4).

## Conclusion

Inspired by the porous structure of fibrous connective tissues, this study investigated underextrusion in FDM 3D printers as a solution to the challenge of bonding soft and rigid materials in the fabrication of hybrid robots. The results of various bonding experiments demonstrate that the underextrusion interface can withstand higher loads than the traditional adhesive methods used in soft robotics. Specifically, with 30% underextrusion, we achieved 106% higher lap shear force and 226% higher peeling force compared to commercial Sil-poxy adhesives when using Ecoflex 00-10. Furthermore, the proposed method provides a better bonding solution when using more viscous rubbers such as Dragon Skin 10, as it can withstand lap shear forces and peel forces that are 68% and 281% higher, respectively, than those recorded when using silicone-based adhesives as a bonding option. Additionally, in pressure tests critical for hybrid pneumatic actuators, our method achieved four times higher pressure and twice the expansion, resulting in improved actuator performance. Moreover, using 3D printing for bonding enables the creation of more complex structures that are difficult to achieve with adhesives.

For future work, since failures often occur at the bonding point in the soft material due to stress concentration, exploring more transitional printing techniques that mimic natural tissues with a gradient in stiffness from rigid to soft materials could be beneficial. We believe that the proposed bonding technique if integrated into direct silicone rubber 3D printing as illustrated in^38^, could further enhance the manufacturing of hybrid robotic systems. Additionally, experimenting with other 3D printing materials, such as ABS and PETG, which are prone to oozing and may provide more anchor points due to unwanted material being deposited, created more fiber points. Therefore, the combination of more fibers at the bonding points with stronger material properties of these materials compared to PLA could improve bonding. Furthermore, incorporating functional materials like conductive or magnetic filaments could enhance the bonding point, allowing for the integration of sensors and actuators and thereby adding new functionalities to hybrid robotic systems. Finally, through our experiments, we observed that the underextrusion structure encapsulated within the silicone rubber retains its flexibility, although its stretchability is altered. Thus, this work can serve as a foundation for developing more effective transitions between soft and rigid structures and even for modifying the mechanical properties of silicone rubber by adjusting the underextrusion parameters.

## Methods

### Biology and inspiration

Dense connective tissue is one of the most important components in most living organisms, thanks to its numerous mechanical functions of support, protection, and bonding between different structures. Depending on the predominance of fibers that compose it, dense connective tissue can be differentiated as fibrous or elastic. Additionally, a further division can be made based on the arrangement of the fibers that compose the tissue. Dense regular connective tissue is characterized by aligned parallel bundles of fibers, which are consequently grouped in an organized fashion, thus conferring anisotropy, as can be seen in Fig. 1a. This tissue type can be found in multiple biological structures, such as ligaments and tendons^27^. On the other hand, an interwoven array of fibers forms the irregular, dense connective tissue, and it can be found in different anatomical areas that are subjected to multi-directional mechanical stresses, such as the pericardium, which is a fibrous inextensible tissue that surrounds the heart. Other than providing mechanical support to the organs, this tissue is also important for the bonding between rigid and soft structures, such as bones and skeletal muscles, which happens in the periosteum, a membrane that covers the outer surface of all bones^27,28^.

For instance, the so-called Sharpey’s fibers, which comprise the external fibrous layer of the periosteum, are able to connect the latter with the bone by directly penetrating the circumferential and interstitial lamellae of the bone tissue. Moreover, the same fibers connect muscle tissue to the periosteum, thus completing the link between the two tissues^27,28^.

### Controlled weaving of microfibers by under-extrusion

In this work, we exploit commercial FDM printing to fabricate micro fibers similar to fibrous connective tissues in nature at any desired location of the printed structure. Specifically, we were inspired by a common issue in the FDM printing process known as oozing, which creates undesired microfibers around the printed component, and it is usually caused by a dirty or partially clogged nozzle traveling in free space in between printing spots. This travel can stretch the liquid contamination at the tip and create a miniature fiber much smaller than the nominal size of the nozzle. On the other hand, similar thinning behavior can be seen in another FDM printing issue called under extrusion, during which the extruder fails to supply a sufficient volume of material to the nozzle, thus resulting in the stretching of the deposited material and thinning of printed lines. Under-extrusion is usually seen as a problem in additive manufacturing as it can lead to gaps or missing layers, thus directly affecting the quality and integrity of the component. Understanding how these fibers are created and exploiting them in a controlled manner can solve one of the major problems found within the soft robotics community, which is related to the bonding between soft and rigid bodies. Achieving good bonding is crucial when manufacturing hybrid systems. For instance, a recurrent problem when conducting this application is related to the bonding between thermoplastics (e.g., PLA) and silicone rubber, which are generally difficult to bond due to their chemical and mechanical incompatibilities.

In this work, we purposely utilized oozing and under-extrusion to create interwoven porous structures that can act as a starting point for similarly bonding different materials to Sharpey’s fibers. This can be achieved by intentionally modifying two main parameters used in additive manufacturing: flow rate percentage and print speed. In the case of commercial PLA filaments, it is generally recommended to employ print speeds between 60 and 80 mm/s and a flow rate of 100–105%, depending on the color of the filament and the model of the 3D printer.

According to the underlying theory of FDM 3D printing, it will be possible to modify the dimensions of a printed filament by purposely changing one or both of the latter two parameters. Following the law of conservation of mass, it is possible to predict the diameter of any molten polymeric filament extruded through a nozzle. When you want to extrude the material over a specific distance ($d_e$), the volume of the material flowing out of the hot nozzle must equal the volume of the raw material fed through the extruder inside of the subsequent nozzle; therefore:

1 $$\begin{aligned} V_{in} = V_{out} \end{aligned}$$

If we hypothesize that the extruded filament has a rectangular section with semicircular ends, we can then determine both variables $V_{in}$ and $V_{out}$ as:

2 $$\begin{aligned} 0.25\pi (D_f)^2 d_{in} = ((l_w-l_h)l_h +0.25\pi (l_h)^2) d_e \end{aligned}$$

With $D_f$ being the diameter of the raw filament, $d_{in}$ is the length of the segment fed through the extruder to extrude over the distance $d_e$. On the other hand, $l_h$ is the chosen layer height and $l_w$ is the actual width of the extruded filament, which we can compute by inverting the previous formula:

3 $$\begin{aligned} l_w = \frac{(0.25\pi (D_f)^2 d_{in})}{(l_h d_e)} - l_h(0.25\pi -1) [mm] \end{aligned}$$

Furthermore, $d_{in}$ is typically calculated by the slicer software depending on the chosen 3D printing parameters:

4 $$\begin{aligned} d_{in} = \frac{\gamma ((l_{wn} l_h d_e)}{(0.25\pi (D_f)^2)} [mm] \end{aligned}$$

The value $l_{wn}$ is the nominal layer width, which is commonly set equal to the diameter of the nozzle, and $l_h$ is the layer height. The factor $\gamma$ indicates the flow rate percentage that can be directly set through the slicer. By substituting the latter expression in Eq. (3), we obtain:

5 $$\begin{aligned} l_w = \gamma l_{wn} - l_h(0.25\pi - 1) [mm] \end{aligned}$$

This should provide us with an approximation of the diameter of the extruded filament depending on its printing parameters, thus allowing us to predict the size of the resulting fibers obtained using the proposed method.

### Theoretical model

**Fig. 5:**
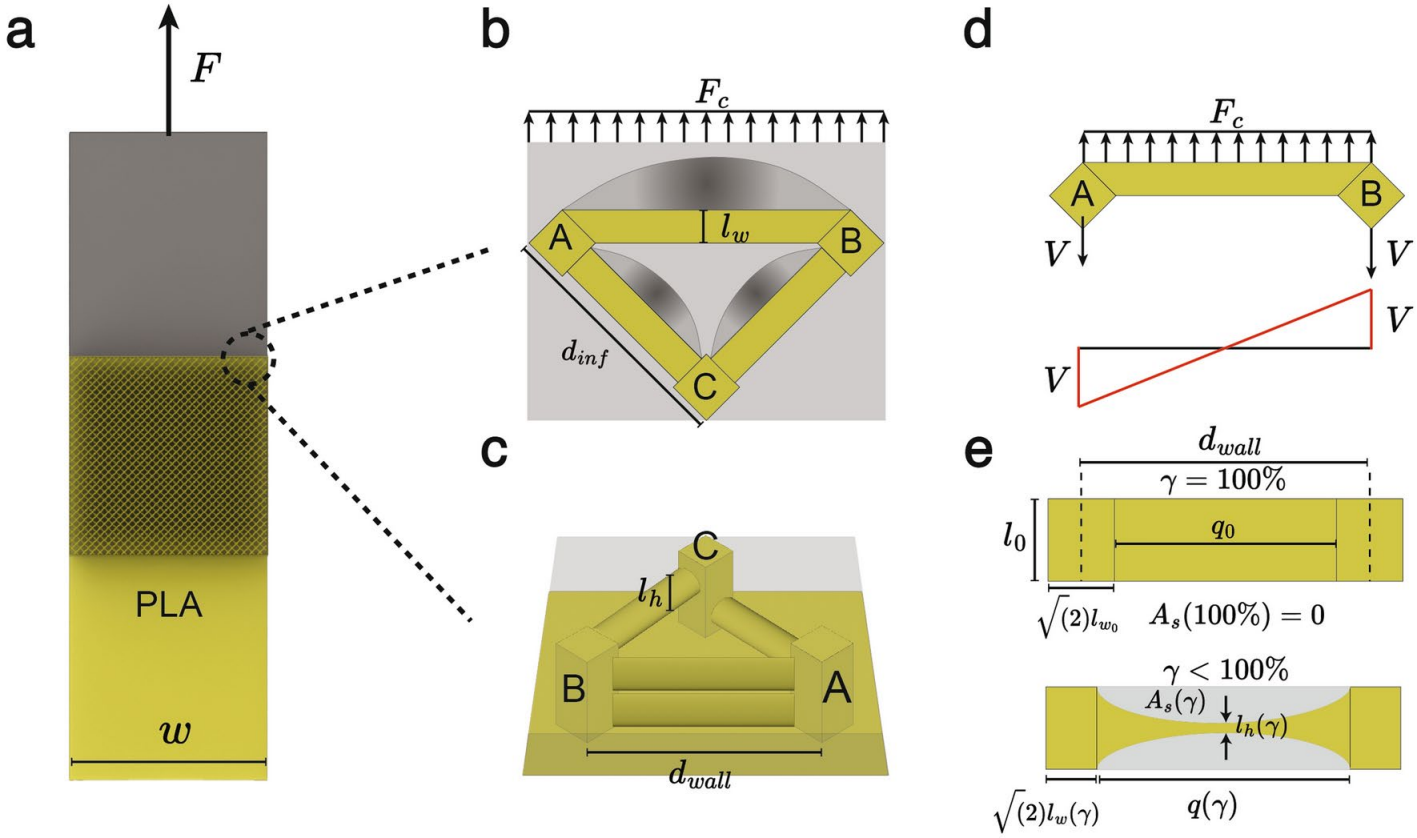
2D rendering of the samples fabricated for the lap shear tests (**a**). The silicone rubber strip (in gray) is under tension at a constant rate. In order to determine a model that allows the estimation of the debonding forces given the chosen infill pattern, we analyzed the behavior under shear of a single unit cell. 2D top view of the unit cell under uniformly distributed load, with the theoretical shape of the deformed beams (**b**). In (**c**), 3D schematics of the same unit cell are shown. Free body diagram of the shear load acting along the wall and on the nodes A and B, which are the points under maximum shear (i.e., 1/2 $F_c$) (**d**). 2D front view of a single printed layer at two different extrusion rates at the wall’s section (**e**). Contrary to the slicer software prediction of a constant layer height along the printing direction, we observe thinning of the printed layer height in accordance with^35^.

Following the same approach detailed in^31^, we developed a theoretical model that allows us to predict the debonding behavior and forces under shear of porous 3D printed samples fabricated with variable flow rates and/or infill percentages. Let’s consider the samples that were created for the lap shear test, as shown in Fig. 5 a, in which the silicone rubber end is subjected to a uniaxial extension at a constant rate. In order to explain what happens to the samples fabricated at different flow rate percentages and infill percentages, it is better to scale down the problem to a single cell (or repetitive unit) of volume dV, as shown in Fig. 5b,c. Considering the printing parameters that were chosen for the samples, in particular the infill geometry (i.e., lines) and the use of an external wall, we identified two possible debonding modes between the two materials. One failure mode is expected to occur as a rupture of the rubber segment under normal tension arising at the interface with the external wall of the printed sample. According to^31^, it is then possible to calculate the maximal cell force at the wall interface by:

6 $$\begin{aligned} F_{c,wmax} = \sigma _{s,\text {ten}} A_s(\gamma ) [N] \end{aligned}$$

where $\sigma _{s,ten}$ is the tensile strength of the silicone rubber (i.e., 0.83 MPa and 2.72 MPa for Ecoflex 00-10 and DragonSkin 10, respectively, as provided by the manufacturers). $A_s$ is the area occupied by the silicone at the wall interface as a function of the extrusion rate $\gamma$. If we consider a unit cell as the one shown in Fig. 5 c, As can be calculated by:

7 $$\begin{aligned} A_s(\gamma ) = (d_{wall} - \sqrt{2}l_w(\gamma ))(l_{h0} - l_h(\gamma )) [mm^2] \end{aligned}$$

where $l_{h0}$ is the layer height set in the slicer, the value of $d_{wall}$ depends on the chosen infill geometry and infill percentage. $l_w(\gamma )$ is the printed line width as a function of the extrusion rate, which can be predicted by 5. A more exhaustive explanation of the provided equations and how they were derived can be found in the Supplementary material. In 6, we introduce a function $l_{h(\gamma )}$ that explains how the layer height varies due to changes in the extrusion rate. Theoretically, every slicer software calculates the layer height of a 3D printed part as constant for all the layers even when the extrusion rate is reduced, as only the layer width is considered to be affected by this parameter. However, according to^35^, and to our printed samples, the layer height is also dependent on the extrusion rate, as shown in Fig. 5e and in the microscopy photo taken on the wall of a sample printed at 30% in Fig. 1c. In Supplementary material, we show how this function was derived based on our experimental evidence. The other failure mode is determined by the rupture caused by shear of the connection points between infill lines and the external wall (i.e., nodes A and B in Fig. 5b,c), which are the points of the cell subjected to the highest shear load shown in the free-body diagram represented in Fig. 5d. Each node can be simplified as beams with a square diamond-shaped cross-section of length $l_w(\gamma )$ fixed to the underlying rigid PLA substrate. The latter failure would result in a complete detachment of the cell unit from the rigid PLA, but it would still be bonded to the silicone rubber matrix. Therefore, according to^31^, the maximum force $F_{c,wmax}$ that can be withstood by an infill-to-wall node (e.g., point A in Fig. 2b):

8 $$\begin{aligned} F_{c,nmax} = \tau _{n,tens} \frac{16(l_w(\gamma ))^2}{9} [N] \end{aligned}$$

where $\tau _{n,tens}$ is the shear tensile strength of the printed material, which, according to the maximum shear stress theory, equals (0.5*110 = 55 MPa) for the PLA used in this study. Once the two forces have been estimated, the minimum between the two values will provide the maximal force sustained by the unit cell before failure^31^. It is important to clarify that the following model was created following different hypotheses, which were stipulated correspondingly to the work in^31^. First, we assume that the load applied to one unit cell is equally and uniformly distributed to all the cells of the printed sample. We are aware that this assumption may not be entirely true, as, in reality, the load distribution may vary, considering the complex hyper-elastic behavior of the rubber. Under this hypothesis, not only can we claim that the failure mode of a single unit cell translates to the debonding behavior of the entire sample, but we can also estimate the forces by which this occurs:

9 $$\begin{aligned} F_{max} = min\{(N_{cells}*N_{layers})F_{c,wmax},N_{cells}*F_{c,nmax}\} [N] \end{aligned}$$

Additionally, we formulated the model based on ideal samples, which are fabricated without any defects or artifacts. Therefore, under this assumption, all the fibers’ sections are characterized by layer height $l_h (\gamma )$ and layer width $l_w(\gamma )$. Additionally, we assume that the height of the silicone rubber is constant, and it equals the height of the porous segment, thus limiting the applied shear load only to the section of the porosity itself. In the case of the infill lap shear test, we had to remove the external walls in order to achieve proper bonding between the silicone rubber and the samples printed at 100% flow. This change brings a modification to the proposed theoretical model, as both the geometry and load distribution of the unit cell change. This leads to a different value of $A_s$, which can be found in the Supplementary material. The calculated $F_{c,wmax}$ is then multiplied by $cos(\theta )$, which is the angle of intersection between the infill lines and the x-axis (i.e., 45$^\circ$ for the ’lines’ infill pattern). Similarly, the value of $F_{c,nmax}$ changes due to the removal of the external wall, as the reaction forces happening on the infill lines connection points will vary.

### Manufacturing approach

All the samples and demonstrators in this work were manufactured using a consistent approach. First, the rigid and under-extruded portions were designed as separate parts of an assembly. Next, we modified the starting code of the printer to utilize a multi-extruder 3D toolchanger capable of printing with multiple materials. When using a multi-material FDM 3D printer, it is typically possible to set different printing parameters for each extruder. By exploiting this feature, we could adjust the printing parameters for different portions of the same component, such as reducing the flow rate percentage for the porous segment. Once the part was sliced, the resulting G-code was modified with a Python script to remove unnecessary steps created by the printer, such as extruder switches, in order to avoid manual cleaning of the code every time a new part needs to be printed.

Once the part is 3D printed and inserted into its specific mold, silicone rubber is cast first onto the under-extruded section and subsequently placed into a vacuum chamber to facilitate the penetration of rubber within the porosity and remove residual air bubbles. Finally, more silicone rubber is added to the mold and subsequently cured, thus completing the manufacturing of the hybrid component.

### Experimental setup and materials

In this work, all the samples and demonstrators were printed with a Creality Ender-5 with a Bowden extruder configuration. The samples were printed with a 0.4 mm nozzle at a 0.2 mm layer height and 210 $^\circ$C. The Supplementary Material provides a more complete overview of the printing parameters.

First, to observe the effect of the extrusion rate on the diameter of the extruded material, we performed microscopy imaging using the Keyence VHX 7000 Digital Microscope. SEM was conducted on a JSM-7200F Field Emission Scanning Electron Microscope. We 3D printed 15 samples of 20 mm x 20 mm x 3 mm of poly-lactide acid (PLA, RS-PRO 1.75 mm) with a printing temperature of 210 $^\circ C$ with a plotting speed of 80 mm/sec. The sample featured a fully extruded bottom layer of 1 mm thickness, with the top 2 mm varying extrusion rates set at 10%, 20%, 30%, 40%, 50%, 60%, 80%, and 100%. In addition, we cast Ecoflex 00-10 (Smooth-ON) into the porous structure of half of the samples to investigate silicone penetration inside the underextrusion. We chose Ecoflex 00-10 since it is a highly soft rubber, popular in soft robotics, and difficult to bond to a rigid substrate.

Furthermore, we performed a lap shear test to evaluate the bonding efficacy between commonly used adhesives in soft robotics, such as Sil-Poxy silicone rubber adhesive (Smooth-on), and our proposed method. The lap shear adhesion test followed the ASTM D5868 standard for fiber-reinforced plastics. In this test, we aimed to analyze how three different flow rate percentages (i.e., 10%, 30%, and 50%) affected the bonding between the chosen printing material (i.e., PLA) and two casting materials of different stiffnesses, Ecoflex 00-10 and Dragon-Skin 10 (Smooth-on).

For this test, a total of 12 samples (three for each flow rate percentage and three for glue) were fabricated by deliberately underextruding a $20 mm \times 20 mm \times 2 mm$ segment of the specimen, as shown in Fig. S3 of the Supplementary Material. A $20 mm \times 20 mm \times 2 mm$ soft strip was subsequently made by casting Ecoflex 00-10 over the printed sample with an overlapping surface equal to the one circumscribed by the resulting porous structure. The test procedure consists of pulling the free segment of the silicone rubber at 13 mm/min with a universal tensile tester (Instron 3343, Instron, USA) until rupture of the sample.

The same test was conducted to study how changing the infill percentage would affect the bond strength between PLA samples printed with the proposed technique at a 30% extrusion rate, in comparison with samples printed at the flow rate recommended by the manufacturer (i.e., 100%). The chosen infill percentages were 25%, 50%, and 100%. For the latter test, a total of 12 samples (three for each combination of samples) were fabricated similarly to the other lap shear test.

In addition, a 180-degree peeling test was conducted following the ASTM D903 standard test to compare the peeling performance for measuring the stripping strength of adhesively bonded materials, as seen in Fig. S3 of the supplementary material. However, due to the limited print bed and the large dimensions in the standard, we scaled down the dimensions and the experiment speed by six times. The influence of the flow rate percentage variation on the bond strength when using the proposed technique was compared to the bond strength recorded using a silicone adhesive. Therefore, the same number of samples as the lap shear test was manufactured for the peel test.

Circular samples with a 50*mm* outer diameter were made for the ballooning pressure tests. A 2*mm* thick external circular crown section with an inner diameter of 40 mm was printed on top of the rigid PLA substrate following our proposed underextrusion method. Similar to the bonding tests, Ecoflex 00-10 and Dragon-skin 10 (Smooth-on) were the chosen casting material in this case. To ensure consistent rubber thickness across all samples, we used a film applicator blade and carefully leveled it over the mold to maintain uniform thickness. This test evaluated the pressure withstood by hybrid inflatable structures manufactured with our method compared to inflatables made using Sil-Poxy$^{TM}$ as an adhesive.

All the samples were inflated with a syringe (Henke Sass Wolf 50 ml) system controlled via a 3D printer driver board (Bigtreetech. Octopus v1.1 3D printer board, eight stepper driver outputs) and the pressures were recorded with MPX5100DP air pressure sensors (NXP Semiconductors, The Netherlands). Through the control driver, we pumped 10ml/sec. At first, the test was conducted until the resulting inflated structure leaked. This was done to determine not only the maximum pressure that the samples could withstand but also the dimensions of the inflated balloons, which were obtained by analyzing the test videos. Moreover, following the initial pressure results, we chose the 30$\%$ sample with Ecoflex 00-10 due to the high deformation and usage of this material in the soft robotics community. We did a cyclic test for 1000 cycles with 80% of the maximum tolerable pressure recorded in the initial experiments.

### Demonstrators design

We developed two demonstrations to showcase the application of rigidity in soft robotics and the usefulness of strong bonding between rigid and soft materials.

First, similar to animals that utilize claws and nails to enhance their manipulation capabilities, we added a 3D-printed nail to facilitate the soft actuators in grasping small objects such as LEDs or nuts. This nail design comprises a rigid component emulating the outer structure of the nail, known scientifically as the nail plate, and an underextruded segment mimicking the connection between the connective fibers of the nail plate, which is made of Keratin (keratinous) and the underlying soft nail bed. Integration of this nail was achieved by slight modifications to the molds of a common fiber-reinforced bending actuator, enabling the insertion of the nail and facilitating one-time casting of the finger to bond with the nail. Such bonding allows for more durability and higher load capacity than merely adhering to the actuator.

In another demonstration, we designed a ballooning gripper with a rigid 3D printed structure, aiming to harness rigid materials’ load capacity while benefiting from soft materials’ adaptability to manipulate various objects. This design comprises panels featuring ballooning actuators on both sides, each cast separately. Subsequently, these panels were interconnected using silicone rubber in a hexagonal configuration to create soft bending joints between the rigid panels. This hexagonal structure not only retains the rigidity and adaptability of the ballooning gripper but also can deform into various shapes to accommodate different objects. Furthermore, utilizing underextrusion instead of conventional adhesives yields higher pressure values, enhancing the gripper’s grasping capabilities.

## Supplementary Information

Supplementary Information 1.

Supplementary Information 2.

Supplementary Information 3.

Supplementary Information 4.

Supplementary Information 5.

## Data Availability

The datasets generated and/or analyzed during the current study are available in the University of Twente, The Soft Robotics Lab, Publication repository, https://tinyurl.com/dk9hxksk. For any additional information, please contact a.sadeghi@utwente.nl.
