# Supplementary material for: Bio-inspired 3D printing approach for bonding soft and rigid materials through underextrusion
Source: Sci Rep. 2025 Feb 5;15:4429. doi: 10.1038/s41598-024-84525-7 (PMC11799307; doi:10.1038/s41598-024-84525-7)
Supplement: Supplementary file 1 — Supplementary Information 1. [file 41598_2024_84525_MOESM1_ESM.docx]

Supplementary Information: Bio-inspired 3D Printing for Bonding Soft and Rigid Material through Underextrusion

Arman Goshtasbi^1,⸸^, Luca Grignaffini^2,⸸^, Ali Sadeghi^2,*^

^1^ SDU Soft Robotics, Biorobotics, The Maersk Me-Kinney Moller Institute, University of Southern Denmark (SDU), Odense M, 5230, Denmark

^2^ Soft Robotics Laboratory, Department of Biomechanical Engineering, Faculty of Engineering Technology, University of Twente, Enschede, 7522NB, The Netherlands

^*^ a.sadeghi@utwente.nl

^⸸^these authors contributed equally to this work

**Measuring the thickness of the printed fiber**

As explained in the Microscopy test and shown in Table 1, we measured the diameter of the printed fiber under different underextrusion percentages using a Keyence VHX 7000 Digital Microscope. The measured thickness of the fiber, as presented in Table 1, closely matched the calculated thickness using Eq5, with a mean absolute error of 7 µm. Supplementary Figure S1 indicates that at 80% and 100% underextrusion, the fibers fused together due to the high printing temperature, making it particularly challenging to measure the fiber thickness at 100%. Consequently, we conducted additional microscopy imaging using grid infill to obtain more accurate measurements of the fiber diameter.

Furthermore, we utilized the JSM-7200F Field Emission Scanning Electron Microscope to verify the repeatability of underextruded layers, as illustrated in Supplementary Figure S2. Finally, we cast Ecoflex 00-10 (Smooth-ON) into the porosity, examined it under the microscope, and observed the penetration of the silicone rubber into the cavity. Figure 1 shows that for underextrusion beyond 60%, the silicone did not fully penetrate the porosity despite being subjected to a vacuum. Due to the surface tension of the silicone rubber, it adhered to the small gaps between the fibers at high underextrusion percentages.

**
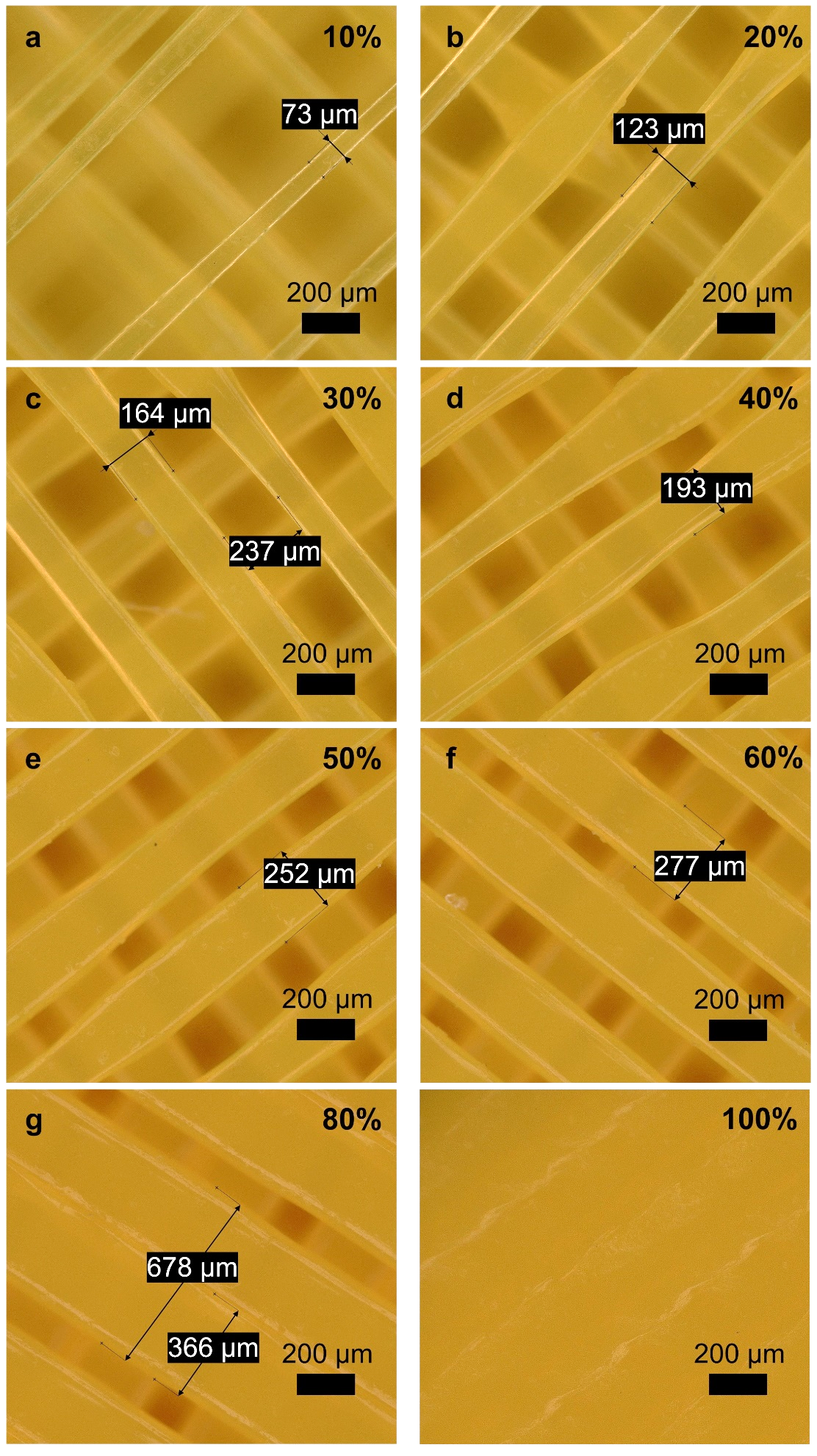
Supplementary Fig. S1**: Optical microscopy of the porous segments of samples 3D printed at different flow rates (all the other printing parameters were the same for all samples; see Table 1 of Supplementary material).


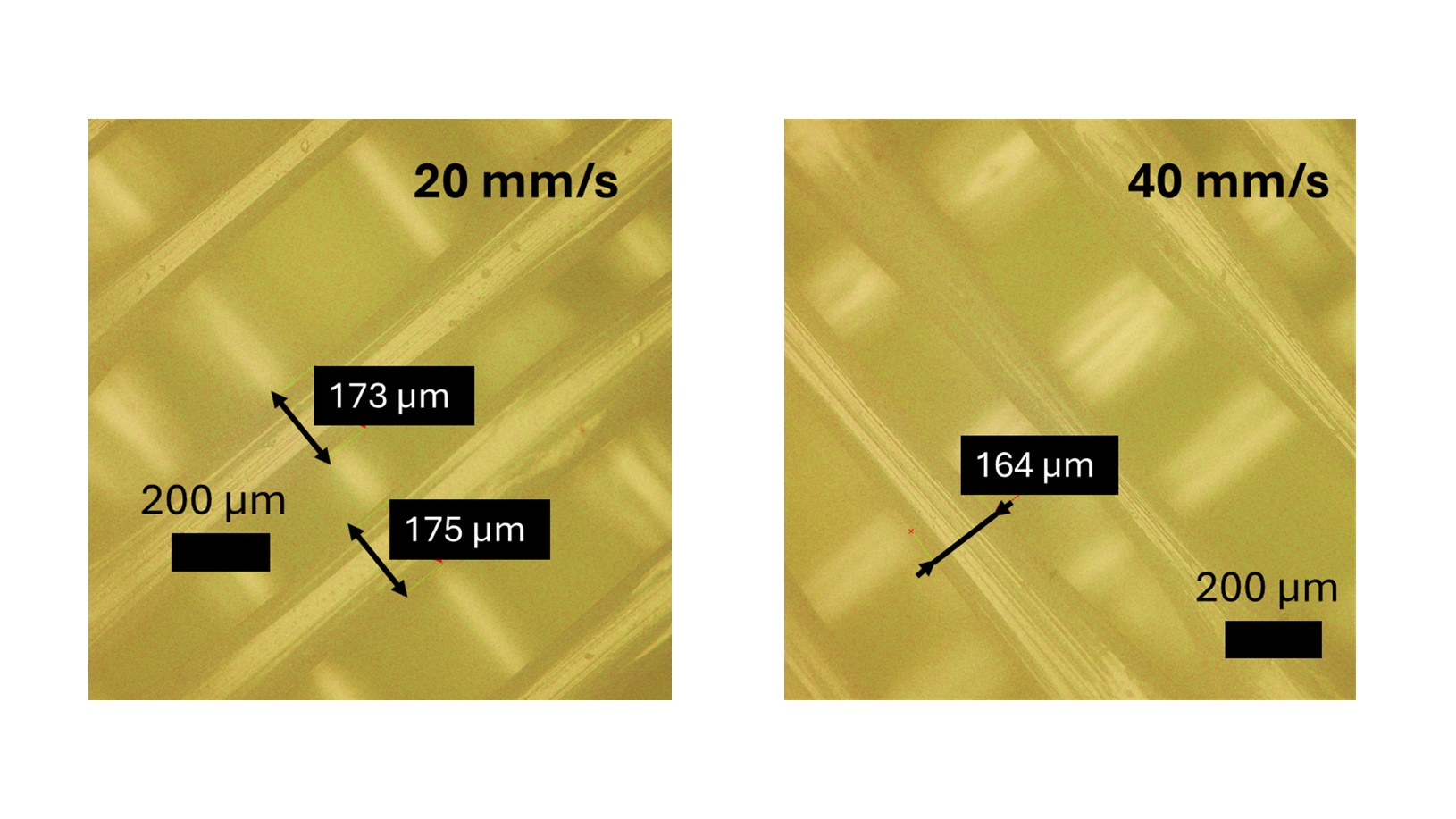
 **Supplementary Fig. S2**: Optical microscopy of the porous segments of samples 3D printed at 30% flow rate and at two different speeds. (All the other printing parameters were the same for all samples; see the section “Printing Parameters” for the supplementary material).


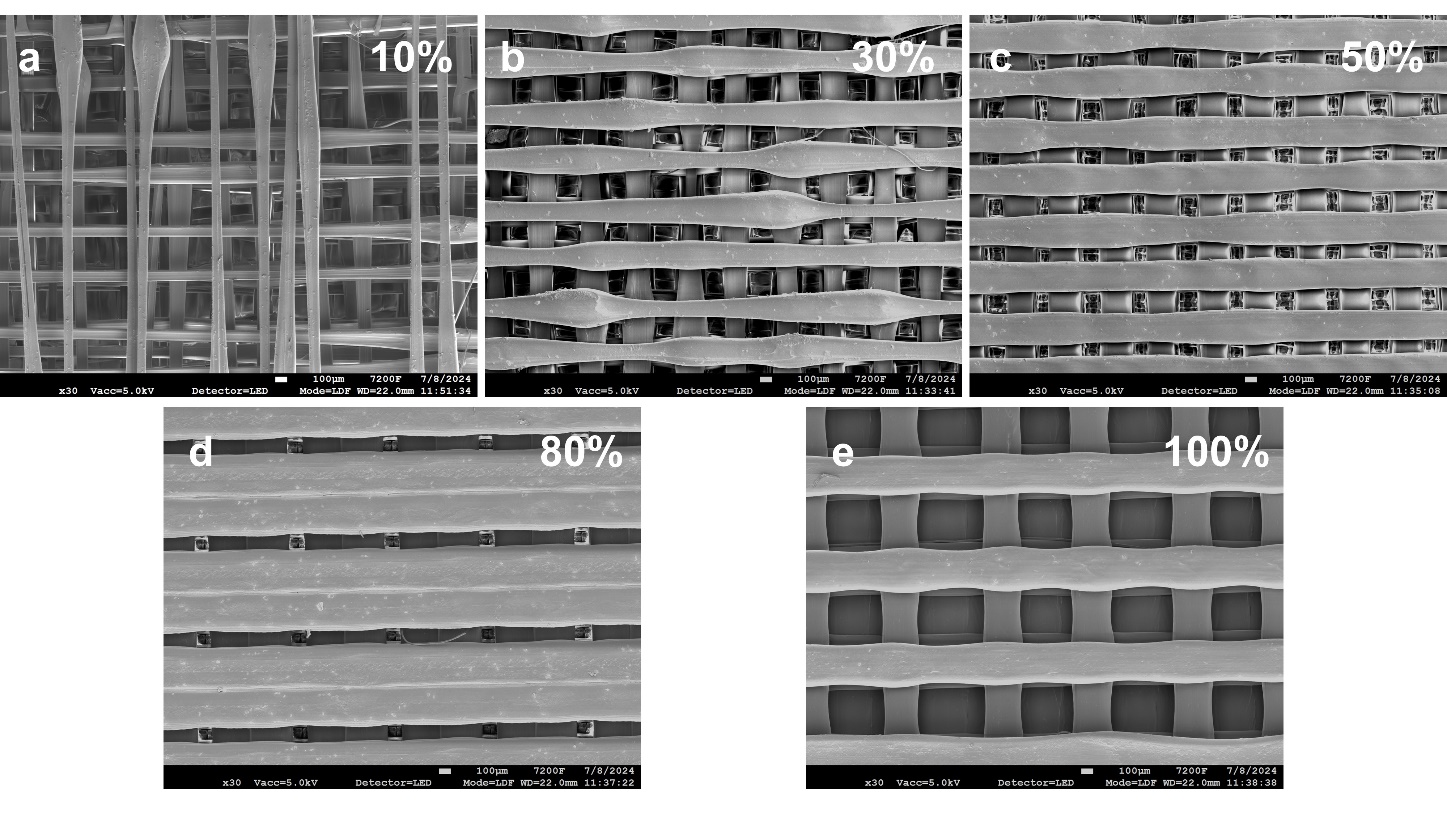
**Supplementary Fig S3**: Scanning Electron Microscopy images of porous segments of samples 3D printed at five different flow rates. The four underextruded segments were captured from the same samples used for Fig.1 (see above), while the sample printed at 100% flow rate was printed at 50% infill in order to measure the diameter of the printed fiber.

**Bonding Tests**

As shown in Fig 2, we conducted a lap shear test and a 180-degree peeling test to compare the bonding of silicone rubber and 3D-printed PLA. This experiment utilized the setup depicted in Supplementary Fig S3. As detailed in the materials and methods section, we modified and rescaled the standard adhesion tests due to the limited bed size of the 3D printer, the lengthy printing times for large objects, and the restricted stroke length of the tensile tester available to us.


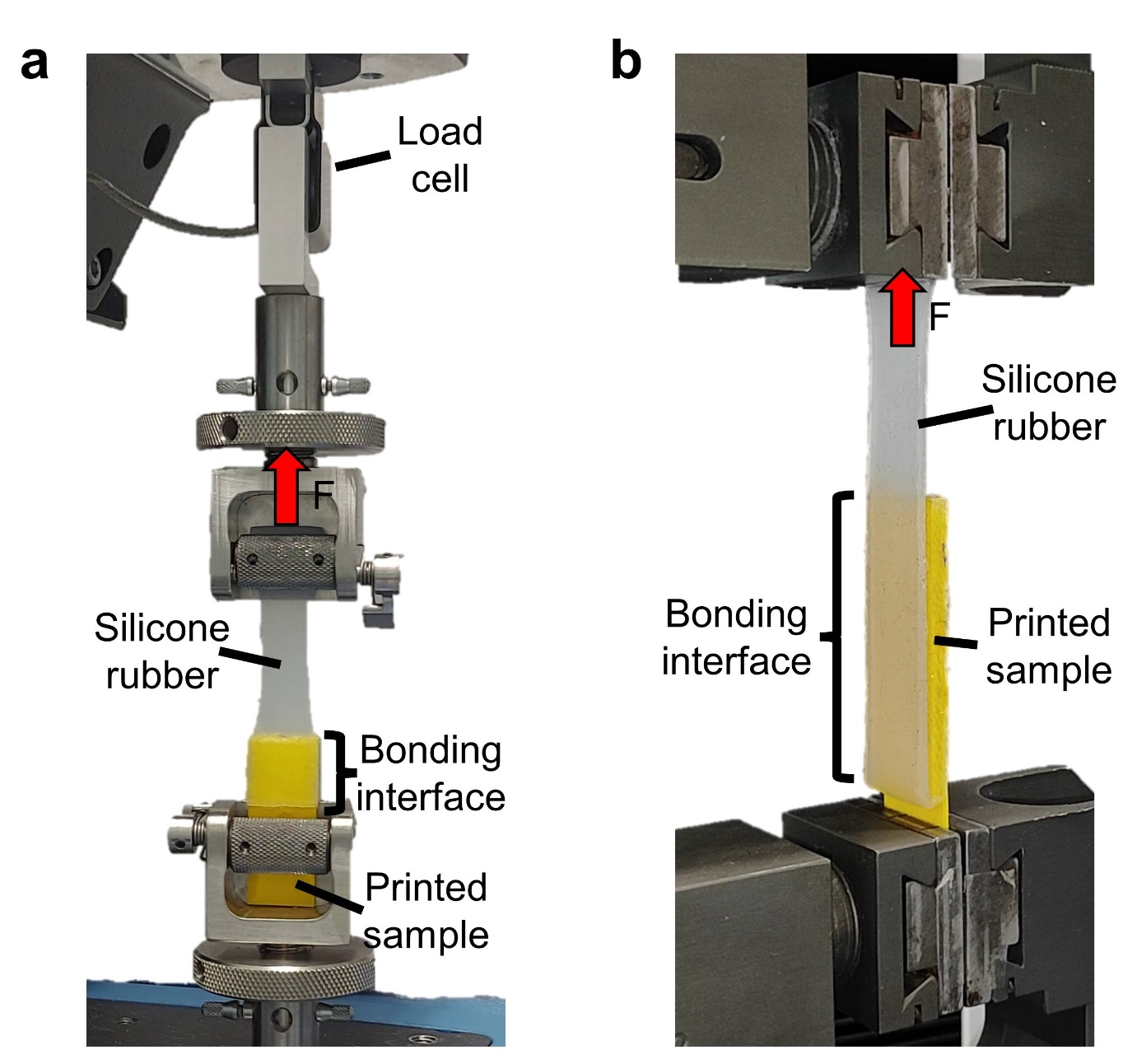


**Supplementary Fig. S4**: Experimental test setup used for the Lap shear test (**a**) and for the 180° peel-off adhesion test (**b**). In both tests, the silicone rubber strip is attached to a load cell and pulled until rupture.

**Effect of speed on the bonding test:**

In addition to the results shown in Fig 2, we performed the same lap shear and 180-degree peeling tests on samples with 30% underextrusion at four different printing speeds. After analyzing the samples at the optical microscope, we saw that the average layer width maintained very similar values (differences of < 10µm) across all printing speeds. Based on this evaluation, we believe that this printing parameter does not affect, as importantly, the bonding behavior. The experimental results proved this, as they were nearly identical across the four printing speeds (20, 40, 60, and 80 mm/sec). However, there were minor variations in the recorded values, which could be attributed to fabrication errors, initial tension during tensile testing, and the way the printer fills and empties the buffer in its system.


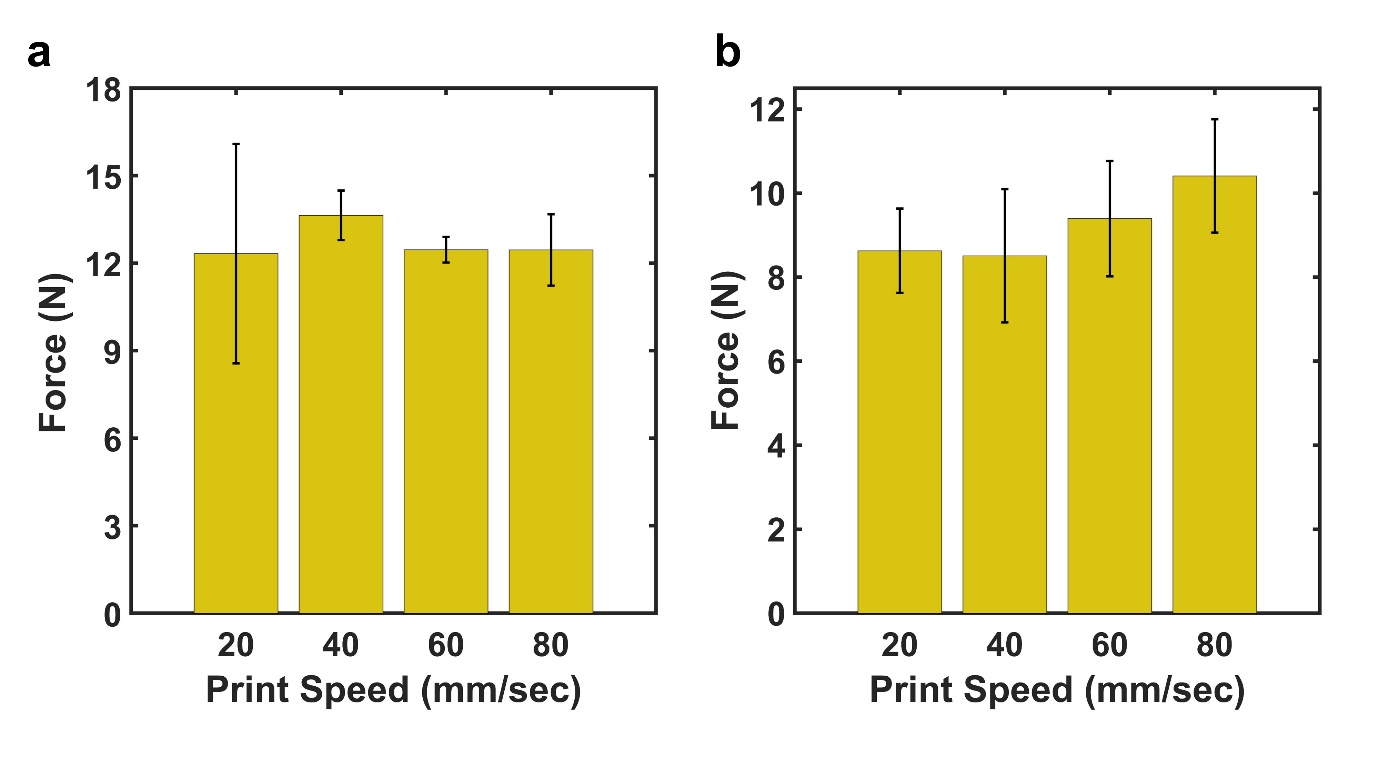
**Supplementary Fig. S5**: Bonding test for samples with 30% underextrusion and Ecoflex 00-10 and different printing speed (**a**) lap shear experiment (**b**). 180 degrees peeling test

**Effect of height of porous segment on the lap shear test:**

In addition to the results shown in Fig 2, we performed the same lap shear on samples with 30% underextrusion at three different heights (i.e., 0.5 mm, 1 mm, and 2 mm) of the porous segment. The silicone rubber strip was maintained at a constant height (i.e., 2 mm) for all three cases. The debonding forces of the sample with a height of 2 mm can be predicted using the theoretical model found in the methods section. In the other two cases, the situation is different, as the load applied on the porosity is more complex (i.e., there is the addition of a dragging behavior of the silicone along the top layer of the underextruded segment) and should be studied further. However, we noticed that the values of the debonding forces were relatively similar across the different samples, as can be seen in Fig S6.


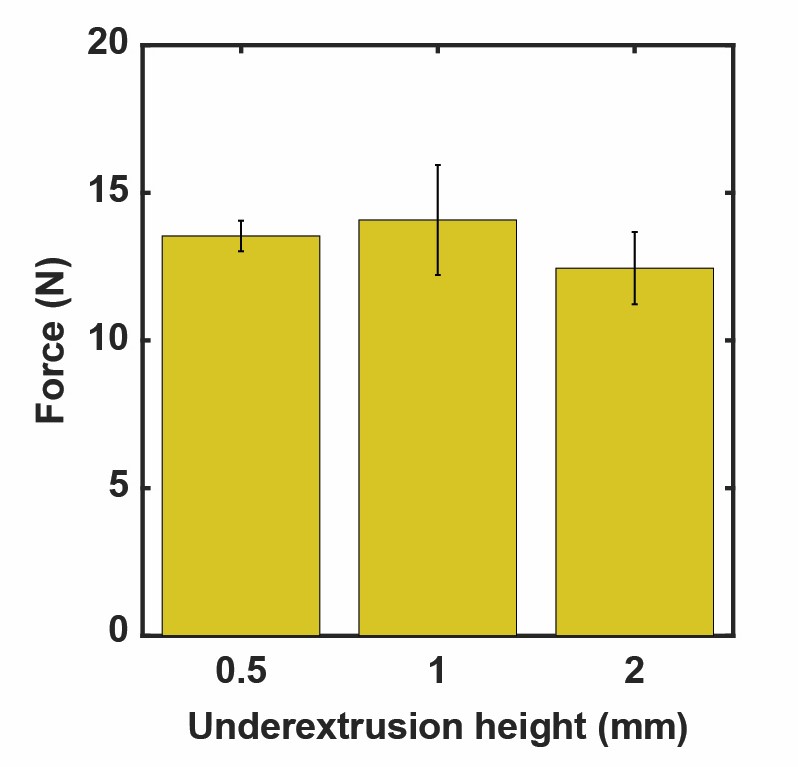


**Supplementary Fig. S6**: Lap shear test for samples with 30% underextrusion and Ecoflex 00-10 and three different porous structure height

**Combining Underextrusion with Sil-poxy:**

In another experiment, we combined our method of creating porous segments through underextrusion with using Sil-Poxy to investigate how underextrusion affects the bonding performance between soft and rigid structures. As illustrated in Fig. S7, adding Sil-Poxy to the underextruded segments significantly enhances adhesion performance compared to its application on the smooth PLA surface. This improvement is anticipated as underextrusion increases the contact area between PLA and Sil-Poxy, resulting in bond failure occurring at much higher loads.


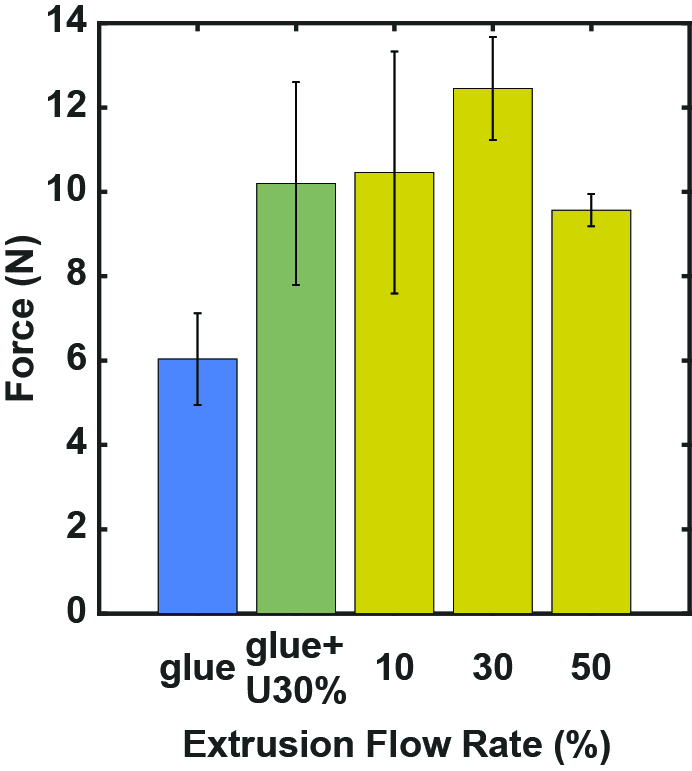


**Supplementary Fig. S7**: Lap shear test for different extrusion rates and compared with glue and glue+underextrusion

**Nail Experiment**

One of the key factors in a nail's ability to pick up objects is its capacity to withstand high axial forces. To investigate this, we conducted experiments similar to the lap shear test, comparing a nail attached to the actuator using Sil-poxy with one connected through underextrusion. The same lap shear test protocol was followed for both setups. Consistent with the lap shear test results, the nail connected via underextrusion demonstrated higher force tolerance compared to the one attached to Sil-poxy.

**
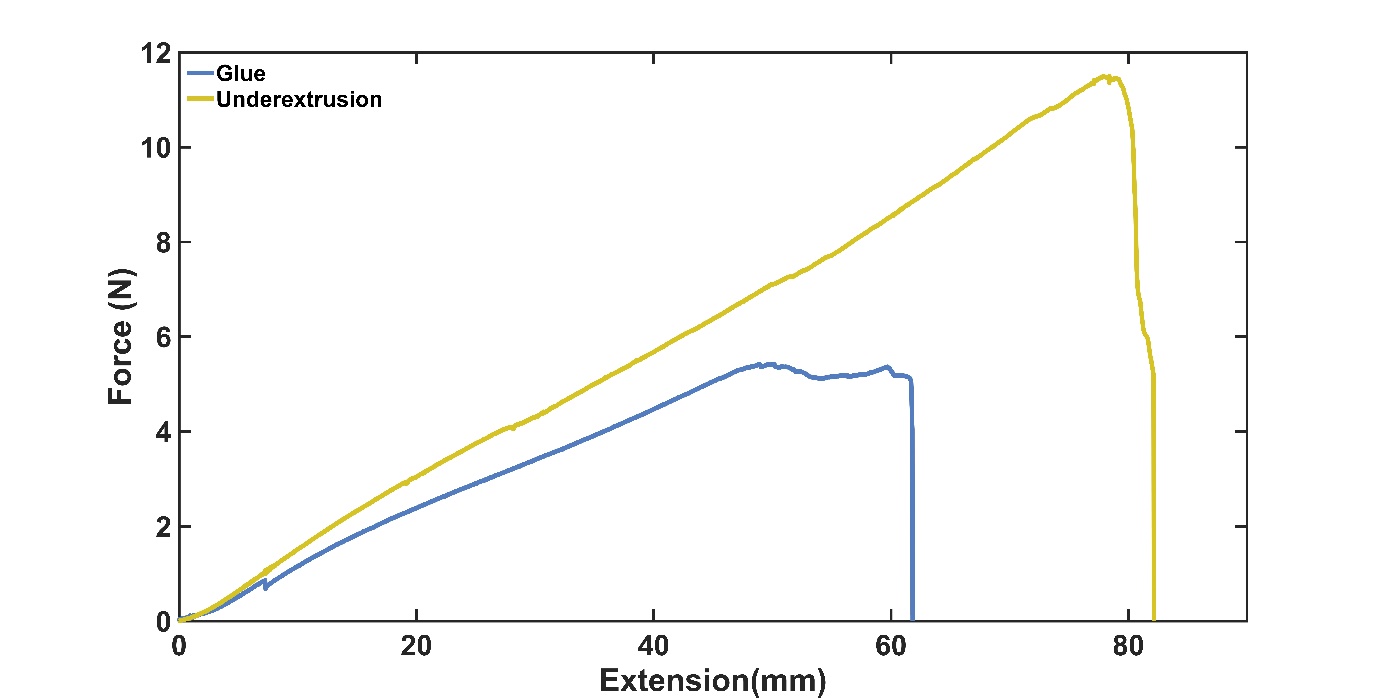
**

**Supplementary Fig. S8**: The bonding strength of the nail attached to the actuator by glue and underextrusion under tensile load.

**Printing Parameters**

To create the G-code for all the samples, we configured a custom FFF (Fused Filament Fabrication) 3D printer profile in Cura 5.3 (Ultimaker, Netherlands). This setup involved defining a dual-extruder system where one extruder was set to print at 100% extrusion, and the other was designated for the underextrusion part of the sample. As detailed in the materials and methods section, each sample was designed separately using CAD software. These designs were then imported into Cura, where the necessary G-code was generated for the 3D printing process. The full parameters can be found as a Cura profile attached to the paper.

Several critical parameters were configured in Cura to ensure the accuracy and quality of the prints. These included:

**Layer Height:** In all samples, we used 0.2mm layer height and 0.2mm initial layer height.

**Nozzle Diameter:** All samples were printed using a 0.4mm brass nozzle.

**Infill:** We only printed with 100% infill in this case to only investigate the porosity created by underextrusion, not the infill and infill pattern.

**Pattern:** We used Lines pattern infill for all samples even the balloon samples to have similar testing for all cases.

**Printing Temperature:** We used RS Components PLA filaments to 3D print all samples, and according to their datasheet, we used 210^o^C to be in the printing temperature range.

**Printing Speed:** Except for the samples made to experiment with the speed effect, all the prints were printed at 80mm/sec.

**Drawings of the Hybrid Gripper**

Here, we present the technical drawings of the Hybrid Grippers proposed in our paper, which were drawn using SolidWorks.

**
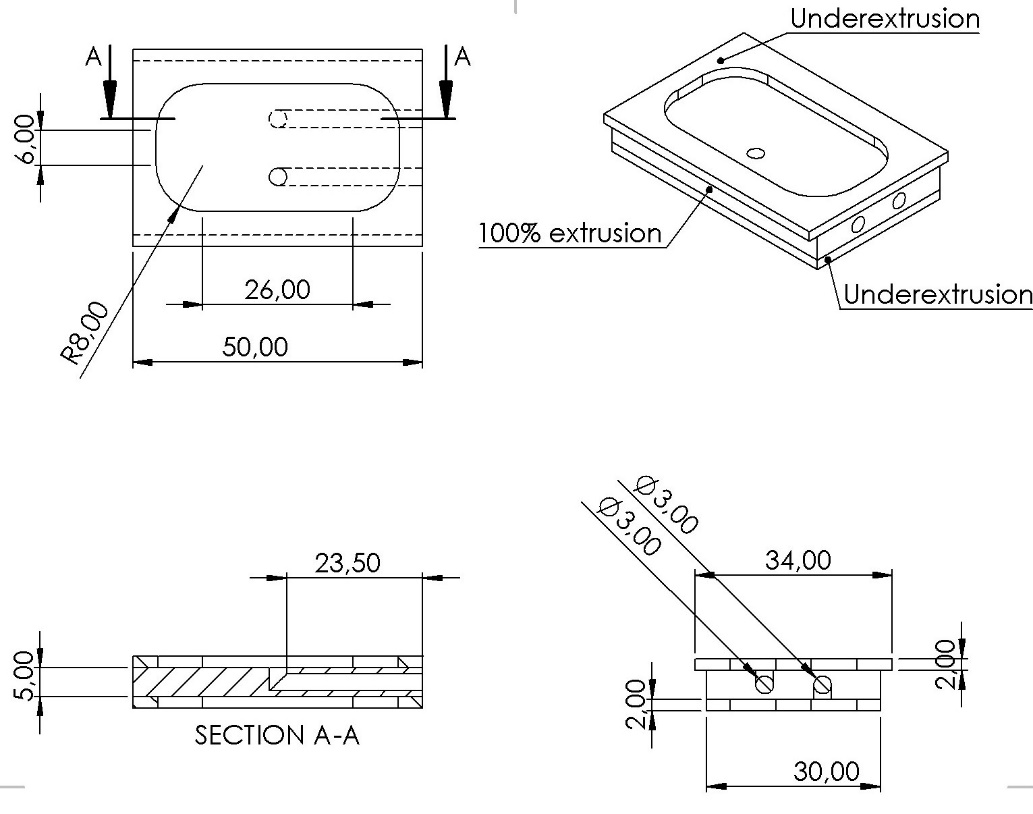
**

**Supplementary Fig. S9**: The drawing of the hexagonal inflatable gripper

**
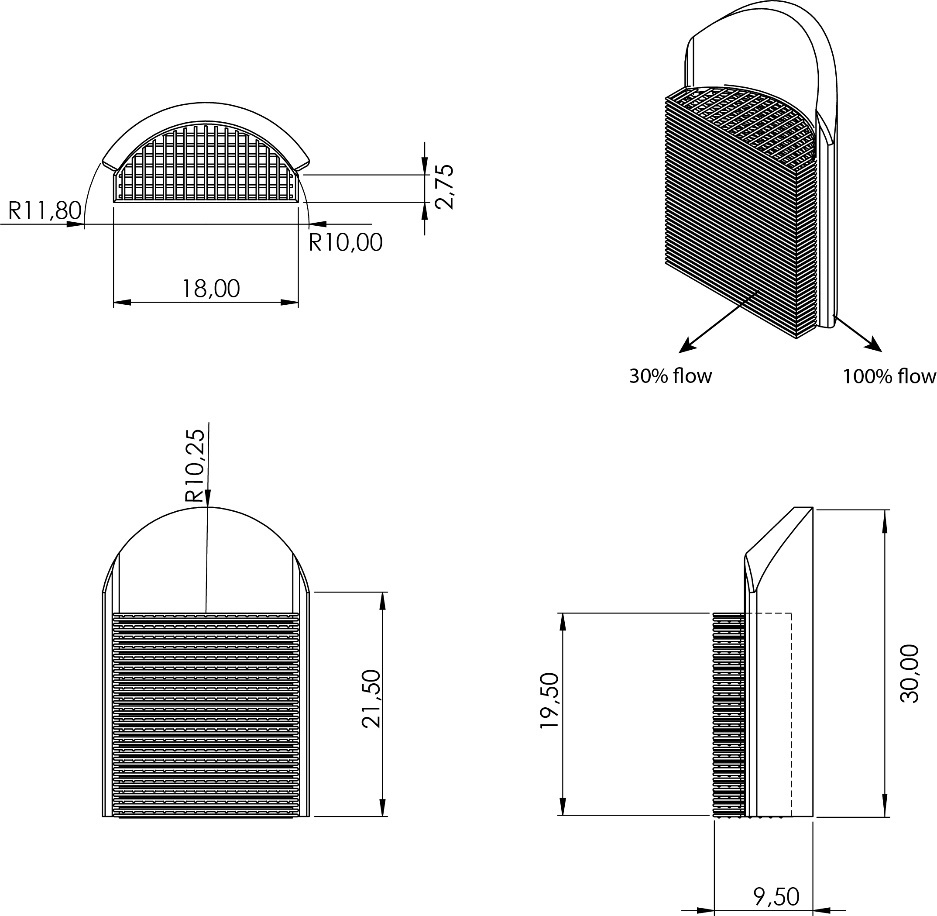
**

**Supplementary Fig. S10**: Nail design for the nail-inspired hybrid gripper

**Supplementary Video S1:**

This video demonstrates the underextrusion printing process in a thin wall at 30% and 50% underextrusion, illustrating how porosity is generated compared to standard 100% extrusion printing.

**Supplementary Video S2:**

This video shows the lap shear test of 10% underextrusion, where the sample breaks by debonding the underextrusion part from its base and peeling tests on 30% samples, where the silicone part breaks while the bonding region remains intact.

**Supplementary Video S3:**

This video illustrates the ballooning of different samples and how significantly the underextrusion improves the possibility of applying higher pressure inside the balloon

**Supplementary Video S4:**

This video showcases the different demonstrators fabricated with this technique and the additional capabilities they provide in soft robotics.
